# A Biobehavioral Validation of the Taylor Aggression Paradigm in Female Adolescents

**DOI:** 10.1038/s41598-019-43456-4

**Published:** 2019-05-07

**Authors:** Lena Rinnewitz, Peter Parzer, Julian Koenig, Katja Bertsch, Romuald Brunner, Franz Resch, Michael Kaess

**Affiliations:** 1 Section for Translational Psychobiology in Child and Adolescent Psychiatry, Department of Child and Adolescent Psychiatry, Center for Psychosocial Medicine, University of Heidelberg, Blumenstrasse 8, 69115 Heidelberg, Germany; 2 Clinic of Child and Adolescent Psychiatry, Center for Psychosocial Medicine, University of Heidelberg, Blumenstrasse 8, 69115 Heidelberg, Germany; 3 Clinic of General Psychiatry, Center for Psychosocial Medicine, University of Heidelberg, Vossstrasse 2, 69115 Heidelberg, Germany; 4 Department of Child and Adolescent Psychiatry, University of Regensburg, Universitätsstraße 84, 93053 Regensburg, Germany; 5 University Hospital of Child and Adolescent Psychiatry and Psychotherapy, University of Bern, Bolligenstrasse 111, 3000 Bern 60, Switzerland

**Keywords:** Psychology, Psychology and behaviour

## Abstract

This research assessed the behavioral, emotional, endocrinological and autonomic reactivity to the laboratory Taylor Aggression Paradigm (TAP) in a sample of healthy female adolescents. Twenty participants were induced with the TAP to behave aggressively (aggression group) and 20 age-matched participants were not induced to behave aggressively (control group). Regression analysis revealed that the aggression group displayed significant higher levels of aggressive behavior compared to the control group (χ^2^ (2) = 255.50, p < 0.0001). Aggressive behavior was not related to self-reported measures of trait aggression, impulsiveness or psychopathy features. Regarding the biological responses, regression analysis on cortisol, missed the set level of significance (χ^2^ (1) = 3.73, p = 0.054), but showed significant effects on heart rate as a function of aggression induction (χ^2^ (1) = 5.81, p = 0.016). While aggression induction was associated with increased autonomic arousal (heart rate), the interpretation of the effects on cortisol warrant caution, given existing differences between groups at baseline and overly elevated cortisol attributable to the general experimental procedures and not the TAP per se. No differences were found with respect to testosterone. In summary, the present study lends preliminary support for the validity of the TAP and its use in female adolescents on a behavioral and autonomic level.

## Introduction

In adolescence, aggressive behavior is associated with distress and impairments in multiple domains, including mental health problems, substance misuse, school drop-out, and suicidal behavior^1,2^. Longitudinal studies examining the developmental trajectories of aggressive behavior indicate life-long negative consequences such as chronic violent and criminal behavior, physical and mental illness, financial problems, and even mortality in adulthood^3–6^.

Research on aggressive behavior in adolescence has predominantly focused on male samples, who have shown to be more aggressive compared to their female counterparts across all ages^7,8^. However, evidence on the increasing prevalence of aggressive behavior among female adolescents^9^ warrants further research. There is evidence that 2–9% of female adolescents meet the diagnostic criteria for conduct disorder^10,11^. Although conduct disorder prevalence in females is lower compared to males, those females with conduct disorder show greater comorbidity and have a less positive prognosis^12^. Another disorder that is highly associated with impulsive-aggressive behavior in female adolescents is borderline personality disorder. Patients with a diagnosis of borderline personality disorder and comorbid antisocial traits show a higher risk for mortality^13^.

Laboratory aggression paradigms are widely used to study social, cognitive and biological mechanisms underlying aggression. One common paradigm is the *Taylor Aggression Paradigm* (TAP), also known as the competitive reaction time task^14^. The TAP allows investigating aggression in an interpersonal situation, where participants compete on a reaction time task against a fictitious opponent. Before each trial, the participant gets to set a level of electric shock to punish the defeated fictitious opponent, and likewise receives electric shocks. The intensity and duration of the electric shocks serve as index for aggressive behavior. Instead of electric shocks recent modifications of the TAP use acoustic tone blasts and add the option to select no punishment^15,16^. Compared to other aggression paradigms, the TAP includes a retaliation of the fictitious opponent, which is close to real-life aggression. Thus, it has been argued that the TAP has increased external validity. Another advantage of the TAP consists of the non-aggressive response option. This limits the participants’ believe that they are expected to show aggressive behavior.

Increasing research investigates the neurobiological underpinnings of aggression, in order to improve our understanding of the genesis of the behavior and ultimately to prevent it. However, little is known about the biological mechanism underlying aggression in female adolescents. Several biological systems have been implicated but not systematically addressed. Stress-regulatory systems including the endocrine system and the autonomic nervous system (ANS) have shown to be associated with aggressive behavior in adolescents^17–21^. Especially, cortisol and testosterone have been proposed to modulate aggressive behavior^20^. Cortisol and testosterone are the end products of the hypothalamic-pituitary-adrenal and the hypothalamic-pituitary-gonadal axes. There are several studies suggesting a negative association between aggressive behavior and cortisol levels in adolescence^21,22^. Beyond cross-sectional evidence, a longitudinal study found lower cortisol levels in preadolescence to be a significant predictor for aggressive behavior five years later^22^. However, other results range from reports on positive associations^23^ to reports on no association between aggressive behavior in adolescence and cortisol at all^24^. Similar to cortisol, studies on testosterone resulted in inconsistent findings^17^. A meta-analysis found testosterone to be weakly related to aggressive behavior^25^. According to Ortiz and Raine^19^ lower resting heart rate (HR) presents the best-replicated biological correlate of aggressive behavior in children and adolescents to date. Adolescents and children with aggressive behavior show lower HR during resting state and in reaction to stress in comparison to healthy and psychiatric controls.

Only a few studies have investigated the biological concomitants of aggressive behavior induced by the TAP. Only one study has specifically addressed the biological reactivity to the TAP within a gender-mixed sample of adolescents^26^. Unfortunately the study made critical changes to the paradigm to measure its respective construct of interest, which did not include aggressive behavior. Further studies exclusively focused on healthy adults and gender-mixed or purely male samples. Most studies measured the neural reactivity in participants undergoing the TAP e.g.^27,28^, but very few studies examined the endocrinological e.g.^15,16,29^ or autonomic e.g.^30^ reactivity to the TAP. With regard to basal cortisol, Böhnke and colleagues^16^ reported cortisol to be negatively related to aggressive behavior on the TAP only in the females, whereas Böhnke and colleagues^15^ found the negative relation to aggressive behavior in both females and males. Acute salivary cortisol levels were significantly higher after the aggression induction by the TAP, when baseline levels were controlled for^15^. Regarding testosterone, healthy males with higher basal levels exhibit more aggressive behavior on the TAP compared to those with lower basal levels^29^. Concerning the autonomic reactivity, in an intoxicated sample a high HR response to alcohol is associated with more aggressive behavior on the TAP than a low HR response to alcohol^30^. Hoaken and colleagues^31^ demonstrated that a non-intoxicated group reacted on the TAP with much greater HR increases than an intoxicated group. But HR response to alcohol did not predict aggressive behavior on the TAP. However, the gap of validation studies displays the further need of research in this area.

These results indicate that performance on the TAP is associated with behavioral, autonomic, and endocrinological responses. Given the lack of previous investigations, the aim of the present study was to provide preliminary evidence on the feasibility of the TAP for the induction and assessment of aggressive behavior in female adolescents. In line with existing research in other populations, it was assumed, that the TAP successfully induces and is capable to measure aggressive behavior in female adolescents. Second, we aimed to investigate if aggressive behavior induced by the TAP is associated with an endocrinological and autonomic signature. Based on previous results, it was hypothesized that the aggression induction would be accompanied by increased cortisol, testosterone and HR.

## Methods

### Participants

The study and the experimental protocols were approved by the ethics committee of the Medical Faculty at the University of Heidelberg and the study was conducted in accordance with the Declaration of Helsinki^32^. Female adolescents, 13–17 years of age, were recruited via flyers and public advertisement. To control for hormonal status, only participants reporting a regular menstrual cycle, no endocrinological disorder, no use of hormonal contraceptives and glucocorticoid medication were considered eligible for inclusion in the study. Adolescents with a current psychiatric diagnosis, any psychological or psychiatric treatment in the last two years, and a history of non-suicidal self-injury or suicidality were excluded from the study. Written informed consent was obtained from participants and their legal guardians. Participants were instructed that the aim of the study was to assess the association between steroid hormones and social relationships. Participants received a compensation of 50€ for their participation. A total of *n* = 69 adolescents were screened for participation and *n* = 29 adolescents were excluded from the study after initial screening. The majority of adolescents interested to participate were excluded due to the intake of hormonal contraceptives (*n* = 14). Five adolescents reported psychological treatment in the last two years or screened positive in the Structured Clinical Interview for DSM-IV Axis I Diagnoses/Non-Patient Edition (SCID-I/NP)^33^. Six participants were finally not interested to participate, three reported no menstrual cycle and one had an endocrinological disease. The resulting sample of 40 adolescents was randomly allocated to one of two groups; an aggression and a control group.

### Procedure

The study comprised two appointments; a structured clinical assessment and the actual experiment. After providing basic sociodemographic data, participants completed the SCID-I/NP^33^ to ensure the absence of any psychiatric disorder. The experimental session was appointed individually for each participant to ensure that saliva samples were collected during the follicular phase of the menstrual cycle. At arrival, participants were introduced to their female opponent (instructed actors). Participants and opponents were seated for approximately 5 minutes in a waiting area and during this time the opponent was instructed to behave neutrally. Participant and opponent were told that they are going to play a competitive reaction time task in separated rooms to avoid reciprocal influence due to facial expression and gestures.

**Figure 1:**
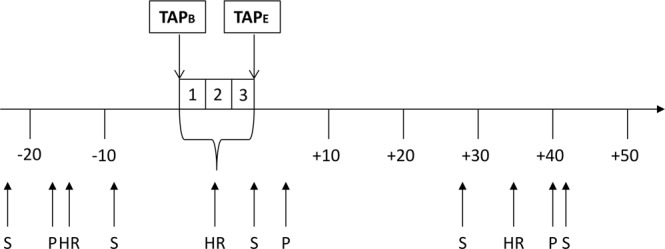
Time line of the experiment session (min). HR = Heart rate, P = PANAS, S = Salivary sample, TAP_B_ = TAP begin, TAP_E_ = TAP end.

Figure 1 provides an overview of the time-flow of the experiment. As the actual time performing the TAP may individually vary, the overview distinguishes between the beginning (TAP_B_) and the ending (TAP_E_) of the TAP. First, participants were attached with the HR monitor. Next, participants provided a baseline saliva sample (T0, −23 min TAP_B_) and completed the Positive and Negative Affect Schedule (PANAS, baseline)^34^. Next, resting HR was recorded for 5 minutes after which participants provided a second saliva sample (T1, −9 min TAP_B_). During the TAP, the experimenter left the room. After completing the TAP, participants immediately provided a third saliva sample (T2, +0 min TAP_E_) and completed the second PANAS. Subsequently, participants completed the Aggression Questionnaire (AQ)^35^, the Youth Psychopathic Traits Inventory (YPI)^36^ and the Barratt Impulsiveness Scale (BIS 11)^37^. Participants were further requested to complete a questionnaire on the whole procedure to validate credibility of the cover story. After that, participants provided a fourth saliva sample (T3, +28 min TAP_E_). Finally, all subjects completed the third PANAS and provided a final saliva sample (T4, +41 min TAP_E_). Participants were debriefed and compensated for their participation. The entire experiment lasted approximately 90 minutes.

### Taylor Aggression Paradigm (TAP)

The TAP was used to induce and measure aggressive behavior^14^. The general procedure of the TAP was adopted from^15,16^. Participants were told that they would play a reaction time task against another participant, the opponent (female actor), who they met and were introduced to before the beginning of the experiment (see above). E-Prime® 2.0 experiment presentation software (Psychology Software Tools, PA, US) was used to present the experimental paradigm on a laptop. The task consisted of 30 trials divided into 3 blocks of 10 trials. In each trial, subjects were instructed to react as quickly as possible to a green square by pressing a key. Participants were made to believe that the loser of a given trial would receive a blast of noise from the winner. Noises were presented through headphones. Before each trial, participants were directed to select the duration and volume of the noise to be presented to their opponent. Noise duration was adjustable between 0 sec (level 0) and 5 sec (level 10) in 0.5 sec increments. Volume was adjustable between 60 dB (level 1) and 105 dB (level 10) in 5 dB increments. With respect to the volume, level 0 represented no noise at all. After each trial, feedback about the outcome of the trial was presented on the screen (i.e., whether the subject won or lost). The ratio of win or lose trials was pre-programmed in the same order for every participant. Each participant won and lost half of the trials. Noise volume and duration were predetermined as well and varied by trial block. A reaction time >1000 ms automatically lead to a loss of the trial in order to support the impression that the participant was playing against a real opponent. During the first block, both groups received short and gentle noises when they lost a trial (volume: M = 62.5 dB, range 0–70 dB; duration: M = 0.75 s, range 0–1.5 s). The control group without aggression induction received the same noises during the second and third blocks. The aggression group received noises of intermediate intensity and duration in the second block (volume: M = 82.5 dB, range 75–90 dB; duration: M = 2.75 sec, range 2–3.5 sec) and of high intensity and duration in the third block (volume: M = 99 dB, range 90–105 dB; duration: M = 4.4 sec, range 3.5–5 sec). The participants’ duration and volume settings were recorded in each trial (0 to 10). An average of volume and duration was computed for each trial. Furthermore, the ten trials within one block were averaged for each participant. These averages represent the dependent variable *aggressive behavior*.

### Endocrinological measures

Endocrinological response to the TAP was measured using salivary cortisol and testosterone. Saliva samples were taken twice prior to the TAP and three times following aggression induction (Fig. 1). Saliva for cortisol analysis was collected using Salivette sampling devices (Sarstedt, Numbrecht, Germany). Saliva for testosterone analysis was sampled in SafeSeal micro tubes (Sarstedt, Numbrecht, Germany), because collection with the Salivette cotton swabs may introduce artifacts in the analyses of testosterone^38^. To limit the influence of diurnal variation on hormonal levels, all experimental procedures and samplings were performed in the afternoon between 3:00 and 6:00 PM^39,40^. Participants were instructed to refrain from drinking and eating for at least half an hour and from physical exercise and smoking two hours before the experiment. Saliva samples were stored uncentrifuged at −20 °C until assay. Cortisol and testosterone levels were assayed at the Department of Psychology of the Dresden University of Technology by using a luminsescence immunoassay, with a lower limit of detection of 1.8 pg/ml for testosterone and 0.276 nmol/L for cortisol. The mean and intra- and inter-assay coefficients of variation were 8% for both hormones.

### Autonomic measures

HR was recorded as marker of autonomic reactivity to the TAP. HR was continuously recorded with a Polar ^®^ RS800CX HR monitor (Polar Electro Oy, FIN) while participants were seated. A strap with electrodes placed to the chest of participants sent wirelessly HR data to the monitor. HR was stored in 5 sec intervals and Polar ^®^ ProTrainer 5 (Polar Electro Oy, FIN) was used to transfer recordings onto a personal computer. Analysis of HR included segments of 5 minutes each, before and after the TAP during resting conditions as well as during the TAP. In the aggression group HR recordings were missing for n = 2 (5%) because of recording failure.

### Positive and negative affect schedule (PANAS)

The PANAS is a self-report instrument that comprises two mood scales, measuring positive (PA) and negative affect (NA). Participants are asked to provide ratings on their current emotional state on 20 items, each rated on a 5-point scale ranging from *very slightly or not at all* to *extremely*^34^. The PANAS was used to investigate whether aggressive behavior induced by the TAP leads to a negative affect. As indicated in Fig. 1, participants completed the PANAS prior and twice following the TAP. Cronbach’s Alpha was α = 0.92 for the PA scale and α = 0.84 for the NA scale.

### The aggression questionnaire (AQ)

The AQ is a self‐report questionnaire designed to assess four dispositional sub-traits of aggression: anger, physical aggression, verbal aggression and hostility. It comprises 29 items that are rated each on a 5-point scale from *extremely uncharacteristic of me* to *extremely characteristic of me*^35^. Internal consistency in the current sample was adequate (Cronbach’s α = 0.91).

### Youth psychopathic traits inventory (YPI)

The YPI is a 50 item youth self-report questionnaire measuring the three core personality dimensions of psychopathy: grandiose–manipulative, callous–unemotional and impulsive–irresponsible. The YPI uses a 4-point scale ranging from *does not apply at all* to *applies very well*^36^. Internal consistency in the current sample was adequate (Cronbach’s α = 0.87).

### Barratt impulsiveness scale version 11 (BIS-11)

The BIS-11 is a self-report questionnaire assessing the personality construct of impulsiveness. The 30 item questionnaire consists of three subscales, including: non-planning, motor and attentional impulsivity. Items are scored on a 4-point scale from *rarely*/*never* to *almost always*/*always*^37^. Internal consistency in the current sample was adequate (Cronbach’s α = 0.78).

The AQ, YPI and BIS-11 were used to test difference in trait aggression and impulsivity between both experimental groups and to verify the convergent validity.

### Statistical analysis

The clinical and sociodemographic characteristics of the two groups were compared with *t-* and χ^2^- tests. Differences by group over time on aggressive behavior, PA, and NA, were tested with multilevel mixed-effects linear regression analyses. Fixed effects were GROUP and the BLOCK of the TAP (aggressive behavior) or TIME point of assessment (PA and NA) respectively, as well as their interaction. Effects on HR, cortisol, and testosterone were determined using mixed-effects linear regression to address differences as a function of aggression induction (cortisol and testosterone at T3/+28 min TAP_E_ in the aggression group in contrast to all other assessments taken independent of group; HR during block 2 and 3 in the aggression group in contrast to the control group and blocks without aggression induction). Fixed effects were GROUP, BLOCK (HR) or TIME (minutes for cortisol and testosterone) and AGGRESSION (cortisol and testosterone: T3/+28 min TAP_E_ in the aggression group; HR: block 2 and 3 in the aggression group). In all regression models the subject ID was used as random effect and Wald tests were used to test the linear contrasts. Sensitivity analyses were made, because graphics showed one outlier in testosterone and two outliers in cortisol. The outliers were characterized by the following z-scores: testosterone *z* = 5.09; cortisol *z*_outlier1_ = 4.34, *z*_outlier2_ = 4.60. As exclusion of the outlier had no relevant influence on the results, the values were included in analyses. The statistical significance level was set to alpha = 0.05. All analyses were conducted using the statistical software Stata 14.0 (StataCorp LP, College Station, TX, US).

## Results

### Descriptive statistics

Sociodemographic characteristics and results from self-report questionnaires by group are provided in Table 1. Group difference were significant (*p* = 0.023) for the subscale BIS nonplanning (not corrected for multiple testing). Since the family wise error for 17 tests at *α* = 0.05 is *p* = 0.582, at least one significant result can be expected, even if the groups do not differ. The p-values range from *p* = 0.331 to *p* = 1.000 when Sidak correction for multiple testing was used. Therefore effect sizes and their 95% confidence interval were reported in Table 1. Variables with effect sizes with *d* ≥ 0.50, reagarding group comparisons, were included as potential covariates in further analyses. As the covariates had no relevant influence on the results, they were not included in the mixed-effects linear regression analyses.

**Table 1:** Demographics and personality characteristics for aggression group (AG) and control group (CG).

	AG		CG		*d*	*CI*	
	*M*	*SD*	*M*	*SD*			
Age	14.85	1.09	15.45	1.00	−0.57	−1.20	0.06
BMI	20.06	2.40	20.68	1.89	−0.29	−0.91	0.34
AQ total	1.95	0.37	1.80	0.37	0.40	−0.23	1.02
AQ anger	2.21	0.48	1.92	0.64	0.52	−0.12	1.14
AQ physical aggression	1.56	0.61	1.52	0.38	0.09	−0.53	0.71
AQ verbal aggression	2.30	0.49	2.18	0.48	0.25	−0.38	0.87
AQ hostility	1.89	0.58	1.75	0.47	0.27	−0.36	0.89
BIS-11 total	65.80	8.46	62.55	7.23	0.41	−0.22	1.04
BIS-11 attention	17.20	3.41	16.30	2.30	0.31	−0.32	0.93
BIS-11 motor	22.70	3.71	22.80	4.51	−0.02	−0.64	0.60
BIS-11 nonplanning	25.90	3.32	23.45	3.24	0.75	0.10	1.38
YPI total	1.80	0.28	1.82	0.30	−0.09	−0.71	0.53
YPI grandiose–manipulative	1.61	0.49	1.71	0.44	−0.22	−0.85	0.40
YPI callous–unemotional	1.66	0.27	1.68	0.31	−0.07	−0.69	0.55
YPI impulsive-irresponsible	2.19	0.36	2.12	0.37	0.19	−0.43	0.82
	***n***	**%**	***n***	**%**	***V***		
School type					0.00		
Gymnasium^a^	14	70	14	70			
Realschule^b^	6	30	6	30			
native language					0.23		
german	20	100	18	90			
other	0	0	2	10			

AG = Aggression group, AQ = Aggression Questionnaire, BIS-11 = Barratt Impulsiveness Scale Version 11, BMI = Body Mass Index, CG = Control group, *CI* = confidence interval, *d* = Cohen’s d, *M* = mean, *n* = cell size, *SD* = standard deviation, *V* = Cramer’s V, YPI = Youth Psychopathic Traits Inventory; ^a^Gymnasium: 8 years of school after 4 years of elementary school terminating with the general qualification for university entry, ^b^Realschule: 6 years of school after 4 years of elementary school terminating with a secondary-school, level I certificate.

### Behavioral measures

**Figure 2:**
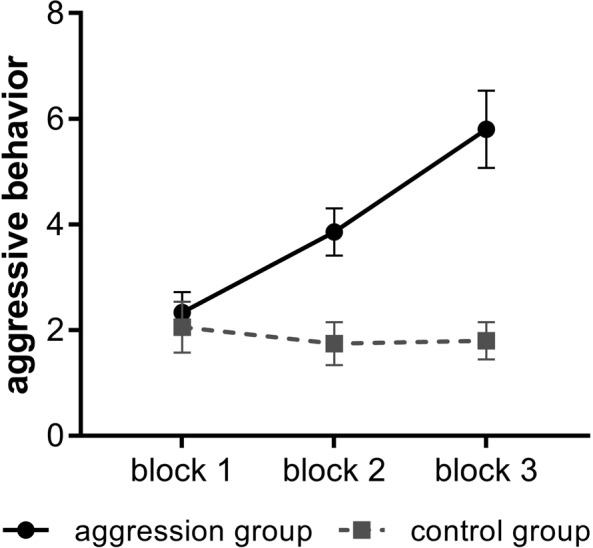
Aggressive behavior by group and time of measurement. Mean aggressive behavior and 95% confidence interval over the three blocks of the TAP in the aggression group (*n* = 20) and control group (*n* = 20).

Means and standard errors of aggressive behavior for each group and block of the TAP are provided in Fig. 2. As expected, regression analysis revealed a significant interaction of GROUP and BLOCK (χ^2^ (2) = 255.50, *p* < 0.0001). Within the aggression group aggressive behavior increased significantly by each block with a linear trend, as displayed in Fig. 2. The control group showed no differences in aggressive behavior between blocks. Accordingly, the aggression group showed significantly more aggressive behavior in block 2 and 3 than participants in the control group. The main effects of GROUP (χ^2^ (1) = 40.39, *p* < 0.0001) and BLOCK (χ^2^ (2) = 187.63, *p* < 0.0001) on aggressive behavior were significant. Correlations showed that aggressive behavior in block 3 was not related to any scale of the AQ, YPI and BIS-11 in the aggression group (−0.20 < all *r* > 0.32, all *p* > 0.166).

### Affective measures

The GROUP by TIME interaction was not significant for PA (χ^2^ (2) = 0.45, *p* = 0.799) or NA (χ^2^ (2) = 0.16, *p* = 0.923). No significant group differences were observed in PA or NA (PA: χ^2^ (1) < 0.01, *p* = 0.967; NA: χ^2^ (1) = 1.92, *p* = 0.166), but there was a significant main effect of TIME on PA (χ^2^ (2) = 57.78, *p* < 0.0001) and NA (χ^2^ (2) = 34.68, *p* < 0.0001). Both groups reported greater NA and PA at baseline compared to the end of the experimental session, with no significant difference in the decrease between groups.

### Endocrinological and autonomic measures

**Figure 3:**
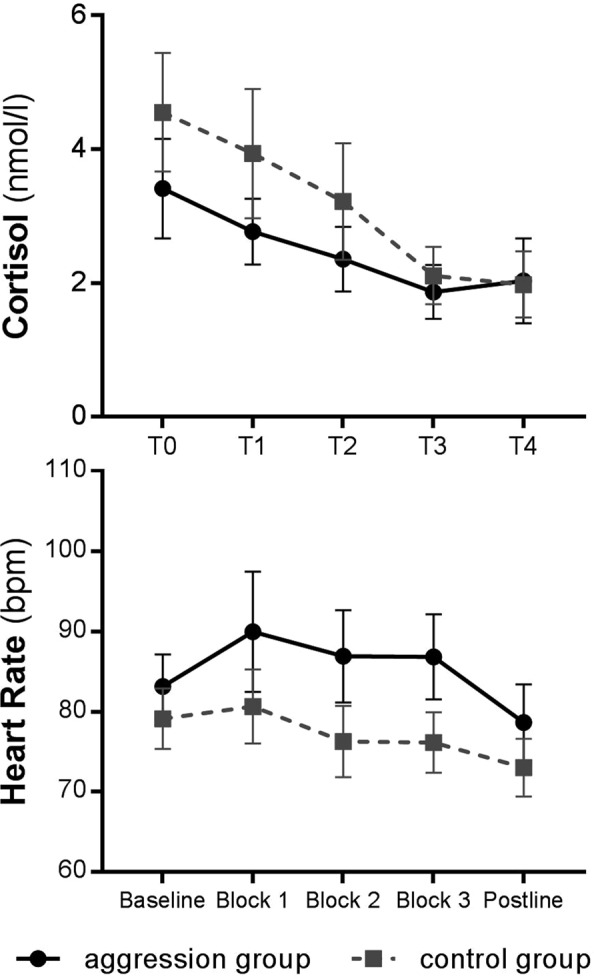
Endocrinological and autonomic measures by group and time of measurement. Mean cortisol levels including their 95% confidence interval in the aggression group (*n* = 20) and control group (*n* = 20). Times of cortisol measurement were 23 min before (T0) and again 9 min shortly before (T1) the TAP_B_, as well as 0 min (T2), 28 min (T3) and 41 min (T4) after the TAP_E_; mean HR including the 95% confidence interval. Differences between the aggression group (*n* = 18) and control group (*n* = 20) in 5 min intervals during resting conditions at baseline and postline, and individually recordings during the TAP.

Regarding cortisol response, regression analyses showed a significant main effect of TIME (χ^2^ (4) = 182.10, *p* < 0.0001). Cortisol levels decreased significantly over time from baseline (T0) over the TAP (T3) to the end of the experimental session (T4) – independent of group. The difference between groups was not statistically significant (χ^2^ (1) = 3.34, *p* = 0.068). Main effects for AGGRESSION (χ^2^ (1) = 3.73, *p* = 0.054) missed the set level of significance. Cortisol levels at T3 (+28 min TAP_E_) in the aggression group did not differ significantly to those of all other assessments taken independent of group. Means and the 95% confidence intervals of cortisol levels over time and for each group are presented in Fig. 3. Correlations showed that the cortisol level at baseline was not related to aggressive behavior in block 3 in the aggression group (*r* = 0.03, *p* = 0.908). Similar, analyses on testosterone showed a significant main effect of TIME (χ^2^ (4) = 39.60, *p* < 0.0001), but no significant main effect of GROUP (χ^2^ (1) = 0.26, *p* = 0.610) or AGGRESSION (χ^2^ (1) = 0.27, *p* = 0.600). Testosterone levels decreased significantly for both groups over the whole procedure, but this decrease did not show a group difference. Regression analysis revealed a significant effect of BLOCK (χ^2^ (4) = 52.58, *p* < 0.0001) and AGGRESSION (χ^2^ (1) = 5.81, *p* = 0.016) on HR. No significant group differences were observed on HR (χ^2^ (1) = 3.71, *p* = 0.054). Post-hoc analyses showed significantly increased HR during AGGRESSION. HR during block 2 and 3 in the aggression group (*M* = 84.32, *SD* = 2.15) was significantly increased in contrast to the control group and blocks without aggression induction (*M* = 80.02, *SD* = 1.63). Effects are illustrated in Fig. 3.

## Discussion

The TAP is a common used laboratory task to induce aggressive behavior. However, regarding its validity and biological concomitants further research is needed. The TAP has only been used in a gender-mixed sample of adolescents^26^ and female samples older than 18 years^41^. The present study aimed to rectify this situation, providing preliminary evidence on the use of the TAP in female adolescents. The primary goal of the present study included the validation of the TAP, addressing behavioral outcomes. Results show that the TAP reliably provokes aggressive behavior in female adolescents. However, the TAP did not trigger a subjective emotional reaction, and we found aggressive behavior unrelated to self-reports of trait aggression, impulsiveness and features of psychopathy in healthy adolescents. Preliminary findings suggest that aggression induction using the TAP is accompanied by a physiological response. While we found evidence that aggression induction leads to an increase in HR, the interpretation of our null findings on cortisol need caution. While analyses missed the set level of statistical significance, visual inspection of the time course of measurements (Fig. 3), highlights potential bias due to group differences at baseline and overly elevated cortisol levels independent of group at the beginning of the experiment. No effects were observed on the testosterone response to the TAP. Further, we found no evidence in support of an association between aggressive behavior and cortisol levels at baseline in the aggression group. However, post-hoc power analyses suggest that whereas the study was sufficiently powered to detect behavioral, affective (PANAS) and autonomic (HR) effects, it may have been underpowered to detect meaningful changes in hormonal concentrations. Power calculations for the endocrinological and affective measures are provided as supplementary information. The autonomic response in the aggression group was characterized by a significant increased HR. One limitation of the study included the problem of multiple testing. As we did not adjust for multiple testing, the results should be judged with caution. In summary, our findings may be carefully interpreted as preliminary support for the validity of the TAP and its use in female adolescents. On a behavioral level, the TAP induced aggressive behavior in the aggression compared to the control group as expected. Findings are supported by differences in the autonomic response between groups – albeit no significant findings for alterations in endocrinological response and affective state.

In line with previous studies^15,16^, the modified version of the TAP used in the present study proves to be a valid paradigm to induce aggressive behavior. Furthermore, and for the first time, results of previous research that were limited to adult and predominantly gender-mixed samples were replicated in a sample of female adolescents. To date there exist only two studies using the TAP in a gender-mixed sample of adolescents. As both studies made critical changes to the paradigm to measure their respective constructs of interest (e.g. influence of executive functioning on verbal aggression^42^, impacts of social context on decision making^26^), they provide no evidence with respect to the general validity of the TAP in adolescent samples.

In response to critical debates about laboratory aggression paradigms^43,44^, the present study used a modified version of the TAP^15,16^. One major deficit discussed in the literature is the lack of a non-aggressive response option in aggression paradigms. Our modified version of the TAP provided participants with the choice to administer no shock. However, future paradigms should include prosocial and communicative response options (e.g. to talk to the confederate, to contact the experimenter, or to leave the setting) to bear resemblance to real world situations. The criticism that the presence of the experimenter might support aggressive behavior was resolved as proposed by Ritter and colleagues^43^. The experimenter left the laboratory for the entire duration of the TAP to allow participants to decide freely. Furthermore, the option of a non-aggressive response alternative limits the participants´ believe that they are expected to show aggressive behavior. Another criticism of aggression paradigms concerns the distance between the participant and confederate, as real-life aggressive conflicts often involves spatial proximity. To improve the credibility of the TAP each participant met her opponent in real life before starting the laboratory session and they were told that they conduct the task in separated rooms to avoid reciprocal influence. To check the credibility of the whole procedure and especially of the TAP participants were asked respective questions regarding the manipulation after debriefing. Responses indicated that all participants trusted the cover. One major criticism the TAP shares with other paradigms is the intention and motivation behind the behavior. Compared to the TAP other paradigms use a provocation before measuring aggressive behavior. Given the provocation, one may discuss if participants’ behavior involves the desire to retaliate rather than to harm. The TAP involves a competitive nature: It has been discussed that participants may administer high shocks or blast noises, because they are primarily motivated to win the task instead of harming the opponent^43,44^. By answering the questions to check the credibility participants reported to experience the blast noise as very unpleasant. In concert with Ferguson and colleagues^45^, we did not find correlations between aggressive behavior and self-report measures of trait aggression. Further, self-report measures of psychopathy features and impulsivity were unrelated to aggressive behavior. This result may be attributed to the healthy sample and the low scores of self-report measures. For the original version of the TAP, good convergent validity has been verified by the positive association between the shock selection and self-reported measures of aggression^46–48^. In accordance to the definition of aggressive behavior from Baron and colleagues^49^, delivering electric shocks is more harmful than administering a blast of noise. But the application of such stimuli in a sample of adolescents would raise serious ethical constraints. Hence an implementation of an alternative punishment (e.g. a blast of noise) is indispensable for the use of the TAP in underage samples. Future studies need to address if the convergent validity of the TAP is reduced when using alternative punishment.

To investigate if the TAP triggers an emotional reaction, the PANAS was used. Both groups reported comparable affective states with a decrease of NA and PA from baseline across the experimental procedure. These results are contrary to Böhnke and colleagues^16^ who showed that adults in the high aggression group reported more negative feelings after the TAP compared to those in the low aggression group. Our results suggest that the manipulation of the TAP is not related to a change in emotional reaction. Since affective states in both groups followed a similar pattern, the present findings do not support the capacity of the TAP induced aggression to alter affective states.

Cortisol, released by the hypothalamic–pituitary–adrenal axis, is the primary component of the body’s endocrine response to stress. On the short term, it can help individuals to recover from stressful experiences and reduce excitability^50^. One might expect that during aggression induction, the aggression group would show greater cortisol secretion in order to regulate stress caused by the aggression induction. While we found no statistically significant differences on cortisol secretion across measurements between groups, the interpretation of this finding warrants caution. Groups showed different baseline levels of cortisol, that independently of group were elevated – potentially due to general effects of the experiment. As illustrated in Fig. 3, the control group showed higher cortisol levels at baseline compared to the aggression group. This may indicate an artifact with heightened endocrinological arousal due to excitement at the beginning of the laboratory session.

Böhnke and colleagues^15^, previously investigated the acute cortisol response to the TAP in healthy adults. Similar to our results, without aggression induction, healthy adults showed a significant decrease of cortisol levels over time, and cortisol levels in response to aggression induction remained relatively stable. Unlike other studies^15,29^, we found no correlation between aggressive behavior on the TAP and cortisol levels in the aggression group. However, studies have shown, that experimentally induced aggression using another paradigm (*Point Subtraction Aggression Paradigm*) correlated positively with cortisol in men^51^. Böhnke and colleagues^15^ explained the divergent results of the two paradigms by the duration. As the *Point Subtraction Aggression Paradigm* included three sessions à 25 minutes compared to 10 minutes duration of the TAP, the Point Subtraction Aggression Paradigm might be more stressful. Furthermore, the study of Gerra and colleagues^51^ included a greater sample size. Nevertheless, no alterations in testosterone levels due to the aggression induction were observed. In contrast, Carré and colleagues^52^ reported an association between aggressive behavior on the *Point Subtraction Aggression Paradigm* and change in testosterone concentrations in men. As women have lower levels of testosterone than men, gender is a widely discussed moderator of the relationship between testosterone and aggression^25^, potentially explaining the present null-finding for testosterone.

Additionally, we found differences in the autonomic (HR) response between groups. In the aggression group HR was significantly increased in contrast to the control group over the course of aggression induction. Thus, one can hypothesize that the aggressive behavior against the opponent produced significant increase in HR. The discrepancy in HR response across the groups lends some supports that the TAP induced aggressive behavior is associated with increased autonomic arousal.

Several limitations should be considered in the interpretation of the present results. First, this validation study included a rather small sample size and we did not adjust for multiple testing. In respect of these major limitations the results of the present study should be judged with caution and require replication. The analyses of correlations within the aggression group were based on a relatively small number of participants and need to be replicated in larger samples. Furthermore, we see several options for improvement and extension of the methodological approach in future studies. We controlled for hormonal status, and participants were instructed to refrain from drinking, eating, sports and smoking for a fixed period before the experiment. Furthermore groups did not differ in age, body mass index and menstrual cycle phase. However, we did not control for pubertal stage and age of menarche onset that have been shown to influence hormone levels^53^. Future studies should address these additional confounders when investigating hormonal reactivity to the TAP. The present study only addressed cortisol and testosterone in association with aggression. However, there is evidence that progesterone and estradiol are also associated with aggression in females depending on the menstrual phase^54^. Greater levels of estradiol and progesterone are suggested to be related to lower levels of aggression in women. Future studies would do well to assess progesterone and estradiol alongside measures of cortisol and testosterone. Another limitation of the study is the fact that groups show different baseline levels of cortisol, that independently of group were elevated. The control group showed higher cortisol levels at baseline in contrast to the aggression group. This may be an artifact attributable to the small sample size and potential confounding variables that were not addressed. Thus, the interpretation of the endocrinological results need caution and require thorough replication. Future research would do well to implement a relaxation period before the actual start of the experiment to disentangle cortisol effects related to the general experimental procedures form those related to aggression induction per se. As the present study used a modified version of the TAP it is not clear, if failed replication of other studies is due to the specific sample or the version of the TAP.

Research on aggressive behavior in adolescence has predominantly focused on male samples, but the increasing prevalence of aggressive behavior among female adolescents demands further research. Accordingly, this is, to our knowledge, the first time that the validity of the TAP was explicitly addressed in a sample of healthy female adolescents. In summary, the study is a first step towards the use of the TAP to investigate female aggressive behavior in adolescence. Future studies in larger samples of female adolescents are warranted to confirm the validity of the paradigm. Experimental research on aggressive behavior faces considerable challenges as the perfect paradigm does not exist yet. Our results contribute to the improvement of experimental aggression paradigms, allowing for the identification of neurobiological correlates of aggressive behavior.

As the ANS and the endocrine system constitute potential biological pathways underlying aggressive behavior, future studies on aggressive behavior should include similar psychophysiological measures. Additionally, the TAP can help to investigate social, cognitive and biological mechanism underlying female-specific aggression in psychiatric disorders, e.g. in conduct disorder or borderline personality disorder.

## Supplementary information

Power calculations of endocrinological and affective measures

## Data Availability

The datasets for this manuscript are not publicly available because of data privacy. Requests to access the datasets should be directed to Prof. Dr. med. Michael Kaess, michael.kaess@upd.ch.
