# Supplementary material for: A Biobehavioral Validation of the Taylor Aggression Paradigm in Female Adolescents
Source: Sci Rep. 2019 May 7;9:7036. doi: 10.1038/s41598-019-43456-4 (PMC6504877; doi:10.1038/s41598-019-43456-4)
Supplement: Supplementary file 1 — Power calculations of endocrinological and affective measures [file 41598_2019_43456_MOESM1_ESM.docx]

A Biobehavioral Validation of the Taylor Aggression Paradigm in Female Adolescents

Lena Rinnewitz ^1, 2^, Peter Parzer ^2^, Julian Koenig ^1^, Katja Bertsch ^3^, Romuald Brunner ^2, 4^, Franz Resch ^2^, Michael Kaess ^1, 2, 5 *^

^1^ Section for Translational Psychobiology in Child and Adolescent Psychiatry, Department of Child and Adolescent Psychiatry, Center for Psychosocial Medicine, University of Heidelberg, Blumenstrasse 8, 69115 Heidelberg, Germany

^2^ Clinic of Child and Adolescent Psychiatry, Center for Psychosocial Medicine, University of Heidelberg, Blumenstrasse 8, 69115 Heidelberg, Germany

^3^ Clinic of General Psychiatry, Center for Psychosocial Medicine, University of Heidelberg, Vossstrasse 2, 69115 Heidelberg, Germany

^4^ Section for Disorders of Personality Development, Department of Child and Adolescent Psychiatry, Center for Psychosocial Medicine, University of Heidelberg, Blumenstrasse 8, 69115 Heidelberg, Germany

^5^ University Hospital of Child and Adolescent Psychiatry and Psychotherapy, University of Bern, Bolligenstrasse 111 3000 Bern 60, Switzerland

**Power calculation of endocrinological and affective measures**

While revising the paper, we were encouraged to provide power calculations for the endocrinological and affective measures as supplemental material. The power calculation for multilevel mixed-effects linear regression analysis poses some challenges^1^. Power analyses were conducted using the SIMR package in R. This is one of the few software solutions allowing power calculations for models with different fixed and random effect specifications^1^. In advance, we specified the effect size for positive and negative affect of the PANAS, cortisol and testosterone. The level of significance for all analyses was set to alpha = 0.05.

**Affective measure (PANAS)**

Each scale of the PANAS includes 10 items each rated on a 5-point scale. Considering the smallest effect that is scientifically meaningful we propose a change of 1 point on the rating scale for half of the items (5). Accordingly, the expected effect size was set. The PANAS was assessed at three different time points. Power calculations always refer to only one parameter in the model. Thus, we decided to focus on the PANAS completed directly following the TAP, because this time point is most meaningful for the validation of the TAP. The present study had a chance of 97.90% and 100.0% respectively, to detect a change in PA or NA as defined. Considering Cohen^2^ the power is above the traditionally acceptable 80%. This leads us to the conclusion that in the absence of statistically significant findings, there were no differences in PA or NA.

**Endocrinological measures (cortisol and testosterone)**

Regarding cortisol and testosterone, we suggested a change of 10% of hormonal concentrations based on the maximum value as smallest, meaningful effect. Both cortisol and testosterone levels were greatest at baseline (T0). Accordingly, the effect size for cortisol was assumed to be 0.5 and the effect size of testosterone 3.0. The power to detect an effect on cortisol as function of aggression induction was 44.90%. Furthermore, there was a 29.50% probability within this study to detect a testosterone effect as a function of aggression induction. The power of approximately 45% indicating a potential Type II error of 55% is under the generally acceptable 80%. The ability to draw a statistical conclusion regarding the cortisol and testosterone change as a function of aggression induction is hampered by the high risk of Type II error. The interpretation of the power calculation of hormones warrants caution, because the estimated effect sizes were smaller than the observed effects. Accordingly, the sample was likely underpowered to detect effects on cortisol and testosterone.

**References**

1. Green, P. & J. MacLeod, C. *SIMR: An R package for power analysis of generalized linear mixed models by simulation*. (2015). doi:10.1111/2041-210X.12504

2. Cohen, J. *Statistical power analysis for the behavioral sciences*. (Erlbaum, 1988).
